# Take a deep BReath: Manipulating brassinosteroid homeostasis helps cereals adapt to environmental stress

**DOI:** 10.1093/plphys/kiaf003

**Published:** 2025-01-06

**Authors:** Karolina Zolkiewicz, Damian Gruszka

**Affiliations:** Faculty of Natural Sciences, Institute of Biology, Biotechnology and Environmental Protection, University of Silesia, 40-032 Katowice, Poland; Faculty of Natural Sciences, Institute of Biology, Biotechnology and Environmental Protection, University of Silesia, 40-032 Katowice, Poland

## Abstract

Global climate change leads to the increased occurrence of environmental stress (including drought and heat stress) during the vegetative and reproductive stages of cereal crop development. Thus, more attention should be given to developing new cereal cultivars with improved tolerance to environmental stress. However, during the development of new stress-tolerant cereal cultivars, the balance between improved stress responses (which occur at the expense of growth) and plant yield needs to be maintained. Thus, the urgent need for developing new cereal germplasm with improved stress tolerance could be fulfilled using semidwarf cereal mutants defective in brassinosteroid (BR) biosynthesis or signaling. BRs are steroid phytohormones that regulate various developmental and physiological processes throughout the plant life cycle. Mutants defective in BR biosynthesis or responses show reduced plant height (dwarfism or semi-dwarfism). Importantly, numerous reports indicate that genetic modification or biotechnological manipulation of BR biosynthesis or signaling genes in cereals such as rice (*Oryza sativa*), maize (*Zea mays*), wheat (*Triticum aestivum*), and barley (*Hordeum vulgare*), which are of crucial importance for global agriculture, may facilitate the development of cereal germplasm with improved stress tolerance. This review presents a comprehensive overview of the genetic manipulation of BR homeostasis in the above-mentioned cereal crops aimed at improving plant responses to various environmental stresses, such as drought, salinity, oxidative stress, thermal stress, and biotic stresses. We highlight target BR-related genes and the effects of genetic manipulation (gene editing, overexpression, and silencing or microRNA-mediated regulation) on plant adaptability to various stresses and provide future perspectives.

## Teaser

A comprehensive review of how manipulating brassinosteroid homeostasis improves tolerance to drought, salinity, oxidative stress, thermal stress, and biotic stress in cereal crops.

## Introduction

The world population is constantly expanding, and according to the United Nations prediction, it will reach 9.8 billion by 2050. Nevertheless, in recent years, worldwide agricultural production has not improved (and has even declined for some crops) as a result of ongoing climate change. If present trends continue, the number of people suffering from food shortage may reach more than 840 million worldwide by 2050. In order to ensure the security of the food supply, global agricultural production needs to double by 2050 (Ahmar et al. 2023a; Tian et al. 2024). Global climate change is associated with the increased occurrence of environmental stresses during the vegetative and reproductive stages of cereal crop development. Environmental stress has deleterious effects on the physiology and yield of cereals (Gupta et al. 2020; Cohen et al. 2021; Baldoni 2022; Eckardt et al. 2023). Cereals constitute an essential global food source. Thus, there is an urgent need to develop stress-tolerant and high-yielding cereal cultivars to provide a sufficient food supply for the world's growing population in the face of ongoing climate change (Ahmar et al. 2024). However, the elite cereal varieties that are currently cultivated were developed mainly for optimal environmental conditions, and their selection was not based on their responses to drought (Ferrero-Serrano and Assmann 2016). Therefore, taking into account ongoing climate change, priority should be given to developing new stress-tolerant cereal cultivars (Fahad et al. 2017; Bechtold and Field 2018; Bailey-Serres et al. 2019). However, it should be kept in mind that during the development of new stress-tolerant cereal cultivars, a balance between improved stress responses (which occur at the expense of growth) and plant yield needs to be maintained (Bechtold and Field 2018; Eckardt et al. 2023).

Enhanced plant survival under drought conditions often corresponds with the constitutive activation of protective mechanisms, such as stomatal closure and osmolyte accumulation, which limit plant growth and result in yield losses. Thus, the tradeoff between drought tolerance and yield is an important aspect that needs to be taken into account (Zhang et al. 2024b). For instance, overexpressing *Grain Weight2* (*TaGW2*), encoding an E3 ligase that regulates grain size in wheat (Zhang et al. 2018b), improved drought tolerance (Li et al. 2024). However, overexpressing *TaGW2* also led to deterioration in yield. The opposite effect was reported after RNAi-mediated knockdown of *TaGW2* (Zhang et al. 2018b; Herrera-Ubaldo 2024). Although several efforts have been made to improve tolerance to drought stress in the model cereal crop rice, only a few cultivars showing improved yield potential and drought tolerance have been developed (Pandey and Shukla 2015). Moreover, overexpressing genes encoding regulatory proteins, including transcription factors from the bZIP, AP2, NAC, and MYB families, improved drought tolerance in transgenic rice plants, but with substantial growth retardation and a negative effect on yield (Todaka et al. 2015). Therefore, finding suitable approaches to achieve the balance between the improved tolerance of cereal crops to environmental stresses and maintenance of yields under various conditions remains an important challenge and requires extensive research.

Among plant metabolic responses to stress conditions, changes in phytohormone metabolism are the most prominent (Zhang et al. 2024b). Brassinosteroids (BRs) constitute a class of steroid phytohormones that play important roles in regulating a wide range of physiological and developmental processes throughout plant life cycle — from seed germination, cell division, elongation, and differentiation to leaf senescence and reproductive development (Ackerman-Lavert and Savaldi-Goldstein 2020; Nolan et al. 2020). BRs also regulate plant responses to diverse (biotic and abiotic) environmental stresses (Tong and Chu 2018). Notably, apart from regulating various developmental and physiological processes, BRs play a crucial role as modulators of plant architecture, and as a consequence, plant responses to environmental conditions (Gruszka 2020). BRs regulate the drought response through (mostly antagonistic) interactions with abscisic acid (ABA), a positive regulator of plant responses to stress conditions (Yoshida et al. 2014; Zhang et al. 2024b). Therefore, the urgent need for the efficient development of new stress-tolerant cereal cultivars may be fulfilled using semi-dwarf, BR-deficient (defective in BR biosynthesis) or BR-insensitive (defective in BR signaling) cereal mutants (Gruszka et al. 2020a).

Genetic manipulation aimed at developing and utilizing new BR-related mutants for the improvement of the yield and environmental adaptability of cereal crops is currently considered to be a target for the next biotechnological revolution in agriculture (Yang et al. 2024). Recent studies have indicated that manipulating BR-related genes using modern genome editing techniques (Clustered Regularly Interspaced Short Palindromic Repeats (CRISPR)/CRISPR-associated protein 9 [CRISPR–Cas9] and HI-Edit) led to substantial improvements in plant architecture, photosynthetic capacity, yield, and nitrogen use efficiency in cereal crops (Song et al. 2023; Tian et al. 2024). Moreover, the BR biosynthesis and signaling processes are considered to be ideal target pathways for biotechnological modification to improve stress tolerance, especially in the face of ongoing global climate change (Gruszka 2020). Importantly, BRs play crucial roles in regulating various traits that are essential for cereal breeding. However, some of these traits are not significantly affected by other phytohormones, including gibberellic acid (GA) (Tong and Chu 2018). For instance, drought suppresses the growth of crop plants, and an increased root-to-shoot biomass ratio represents an adaptation to this stress (Rao and Chaitanya 2016). Therefore, the semi-dwarf BR mutants of cereals may be regarded as preadapted to drought stress through the stress-avoidance strategy (Muller et al. 2011; Dockter et al. 2014; Gruszka et al. 2020b). Furthermore, modulating the BR biosynthesis and signaling pathways could facilitate the development of cereal crops with improved drought tolerance (Jogawat et al. 2021). However, it should be kept in mind that the BR-dependent regulation of the tolerance of cereals to environmental stresses is only partly understood and requires further research (Yang et al. 2024).

In this review, we present a comprehensive overview of the genetic manipulation of BR homeostasis in the crucial cereal crops rice, maize, wheat, and barley to improve tolerance to various environmental stresses, such as drought, salinity, oxidative stress, thermal stress, and biotic stress. We also discuss BR-related genes that became (or may become) targets of genetic and biotechnological manipulation (gene editing, overexpression, silencing, or microRNA [miRNA]–mediated regulation), the effects of this manipulation on adaptability to various stresses, and future research perspectives. Finally, we discuss the recently reported responses (and in some cases improved tolerance) of BR-deficient or BR-insensitive cereal mutants to various environmental stresses.

## Staying alive—reactions of cereal BR mutants to various abiotic stresses

### Cereal BR mutants show improved tolerance to drought

**Figure 1.**
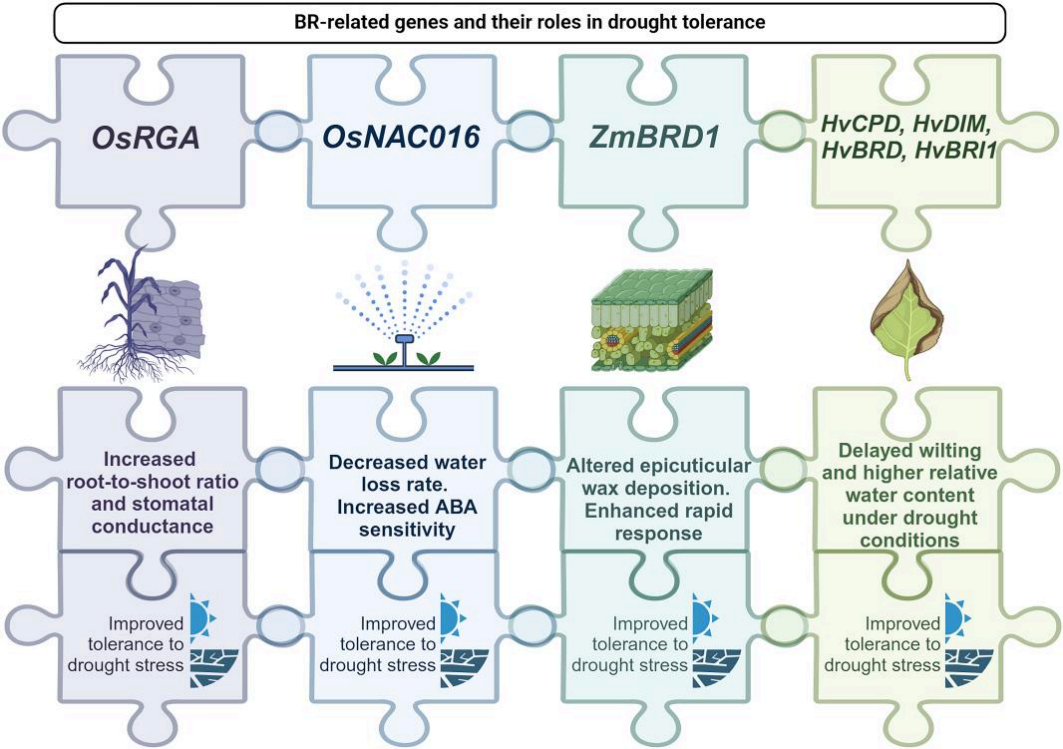
Effects of manipulating BR biosynthesis and BR signaling genes in rice, maize, and barley on the regulation of drought tolerance in these crop species. Full names of the cereal genes are given in the text. Created with BioRender.com. Os, *O. sativa*; RGA, α-subunit of the rice heterotrimeric G protein; *NAC016*, gene from the NAM, ATAF, and CUC family; Zm, *Z. mays*; BRD1, brassinosteroid-deficient dwarf1; Hv, *H. vulgare*; CPD, constitutive photomorphogenic dwarf; DIM, DIMINUTO; BRD, brassinosteroid-6-oxidase; BRI1, brassinosteroid-insensitive1.

The semi-dwarf, erect stature of numerous BR mutants of cereals is a beneficial phenotype that could form a basis for developing novel, stress-tolerant cultivars better adapted to changing environmental conditions, as stress tolerance in plants is often associated with reduced plant growth (Bechtold and Field 2018). One of the first reports on the response of semi-dwarf, erect cereal mutants to drought describes the rice *d1* mutant, which carries a knockout mutation in the gene encoding the α-subunit of heterotrimeric G protein (RGA), which participates in the BR response (Oki et al. 2009; Ferrero-Serrano and Assmann 2016). The first intriguing observation in this study was that the *d1* mutant maintained net photosynthesis for an additional week under drought stress conditions compared with the reference cultivar. Interestingly, during drought, the *d1* mutant exhibited higher stomatal conductance than the reference cultivar, although both genotypes showed similar water loss per leaf area unit. These intriguing features of the response of the *d1* mutant to drought may be explained by its erect stature, which allows for a lower leaf surface temperature. Notably, under drought conditions, the root-to-shoot biomass ratio increased more substantially in the *d1* mutant than the wild type (Ferrero-Serrano and Assmann 2016), indicating that this mutant is better adapted to stress conditions through the above-mentioned stress-avoidance strategy (Muller et al. 2011; Rao and Chaitanya 2016) (Fig. 1). Thus, it was postulated that rice mutants with reduced plant height and erect architecture show improved tolerance to drought and may be used in future breeding programs aimed at developing new, drought-tolerant rice cultivars (Ferrero-Serrano and Assmann 2016). An interesting observation, taking into account the role of glycogen synthase kinase (GSK) proteins in regulating BR signaling (Zolkiewicz and Gruszka 2022), was that a loss-of-function mutation of *OsGSK1* in rice improved tolerance to multiple stress factors, including cold, heat, salt, and drought stress. These findings indicate that the OsGSK1 kinase is a negative regulator of the responses of rice to these abiotic stresses (Koh et al. 2007). Therefore, genes encoding these BR signaling components in cereals represent potential targets of genetic manipulation to develop germplasm with improved abiotic stress tolerance (Choudhary et al. 2012).

The role of the *OsNAC016* gene (from the NAM, ATAF, and CUC family) in regulating rice plant architecture and the drought response was recently proposed. *OsNAC016* encodes a transcription factor that forms a point of interconnection between the BR and ABA signaling pathways, which regulate plant architecture and the drought response, respectively (Wu et al. 2022). The OsNAC016 transcription factor positively regulates the expression of the BR biosynthetic gene *Cytochrome P450 90D2* (*OsCYP90D2*) by direct binding to its promoter to influence plant architecture. On the other hand, OsNAC016 represses the drought response by suppressing ABA signaling (Ahmed and Chen 2022). Interestingly, loss-of-function mutations of *OsNAC016* (introduced through T-DNA insertion and CRISPR/Cas9-mediated editing) resulted in semi-dwarf, erect plant stature, and improved drought tolerance due to decreased water loss rate and increased ABA sensitivity and response (Wu et al. 2022) (Fig. 1). Notably, the OsNAC016 transcription factor interacts with and is phosphorylated by the GSK2 kinase (a negative regulator of BR signaling) and an osmotic stress/ABA-activated protein kinase (SAPK8) that belongs to the sucrose nonfermenting-1-related protein kinase (SnRK2) family involved in ABA signaling (Kobayashi et al. 2005; Wu et al. 2022). Both the GSK3- and SAPK8-mediated phosphorylations of OsNAC016 are required for its ubiquitination-dependent degradation. Thus, OsNAC016 is an important player in the antagonistic interaction between the signaling pathways of these 2 phytohormones (Wu et al. 2022). Moreover, given the semi-dwarf, erect phenotype of the loss-of-function *osnac016* mutants, which results from impaired BR homeostasis and improved drought tolerance, the *OsNAC016* gene may become another target of genetic manipulation to develop new stress-tolerant cereal cultivars.

Studies on BR mutants conducted in maize have mainly focused on the regulation of various developmental processes. However, response of the BR-deficient, dwarf mutant *lilliputian1-1* (*lil1-1*), developed through transposon tagging, to drought stress was also examined in this cereal species (Castorina et al. 2018). *lil1-1* is an allele of the *brassinosteroid-deficient dwarf1* (*Brd1*) gene, which encodes a BR C-6 oxidase involved in BR biosynthesis (Makarevitch et al. 2012). Interestingly, the *lil1-1* mutation, apart from its impact on plant height, stature, and some other phenotypic traits, causes changes in epicuticular wax deposition and improved tolerance to drought at the seedling stage. Moreover, upon rehydration, the mutant plants showed more rapid recovery than the wild type (Fig. 1). This phenomenon may be caused by the better capacity of the mutant plants to retain water due to greater leaf thickness, lower stomatal index, and altered epicuticular waxes. Interestingly, the *lil1-1* mutation does not affect the morphology, weight, or production of maize kernels (Castorina et al. 2018).

The reactions of semi-dwarf, BR-deficient mutants to drought were also analyzed in barley. Two semi-dwarf mutants (carrying missense substitutions) of *HvDWARF*, a gene encoding C6-oxidase, which is involved in the last steps of BR biosynthesis, were exposed to drought at the stage of vegetative development. Interestingly, although under control (optimal watering) conditions, the development (time of heading) of the semi-dwarf BR mutants was delayed (by 20 d) compared with the reference cultivar (“Delisa”), under drought conditions, all tested genotypes (including the reference cultivar) showed similar heading times (Janeczko et al. 2016). This is an intriguing and important observation, as it is generally thought that delayed reproductive development is a drawback of semi-dwarf BR mutants in cereals, which usually show delayed senescence and flowering under optimal watering conditions (Domagalska et al. 2007; Liu et al. 2023). This phenomenon was not observed in the semi-dwarf BR barley mutants under drought conditions. Perhaps drought stress extended the time of heading in the reference cultivar (compared with control conditions), whereas in the semi-dwarf BR mutants, drought stress brought about a decrease in the time of heading (compared with control conditions), resulting in similar heading times among all tested genotypes. Importantly, the semi-dwarf BR-deficient mutants were also less affected by the drought-related growth reduction than their parent cultivar. Drought stress caused a significant decrease in plant height in the reference cultivar, whereas the semi-dwarf, BR-deficient mutants under both (control and drought) conditions showed very similar plant heights. This phenomenon was observed under drought stress and after the rehydration phase (Janeczko et al. 2016). Perhaps due to their semi-dwarf, erect architecture, the semi-dwarf BR mutants are preadapted to drought stress through the stress-avoidance strategy (Muller et al. 2011; Rao and Chaitanya 2016).

Another study was conducted in barley to investigate whether the increased tolerance of the semi-dwarf barley BR mutants to drought is dependent on the genetic background of the analyzed genotypes. In this experiment, 3 semi-dwarf Bowman near-isogenic lines (NILs) deficient in BR biosynthesis and carrying mutations in the *DIMINUTO* (*HvDIM*), *Constitutive Photomorphogenic Dwarf* (*HvCPD*), and *brassinosteroid-6-oxidase* (*HvBRD*) genes and 2 NILs defective in BR perception due to mutations in gene encoding the brassinosteroid-insensitive1 (HvBRI1) receptor, including the original *uzu1.a* allele, along with the reference “Bowman” cultivar, were exposed to optimal watering (control) and drought conditions during vegetative development. Phenotypic observations and physiological analyses indicated that plants of the analyzed semi-dwarf NILs exhibited delayed wilting compared with the “Bowman” cultivar. Symptoms of leaf wilting were first observed in the “Bowman” cultivar, whereas the NILs primarily manifested leaf blade rolling along the longitudinal axis, which may be regarded as a mechanism to reduce transpiration. The study confirmed that the observed enhanced tolerance of the semi-dwarf barley BR mutants to drought is independent of the genetic background of the analyzed genotypes (Gruszka et al. 2016). In another study, the semi-dwarf BR barley mutants exhibited delayed wilting and maintained higher relative water content under drought conditions compared with the reference cultivar (Fig. 1). This is likely due to a reduced demand for water or better redistribution of water within the semi-dwarf plants under stress conditions (Gruszka et al. 2020b). Notably, the perturbation of BR biosynthesis or signaling had no negative (deteriorating) effect on the contents of chemical components in barley grains. Under both optimal watering conditions and upon exposure to drought, grains of the analyzed NILs contained similar or higher contents of dry mineral matter, fiber, proteins, starch, and lipids compared with “Bowman” grains. This is an important issue in light of the potential application of the semi-dwarf barley BR mutants as materials in future breeding programs, particularly in the face of ongoing global climate change (Gruszka et al. 2020a).

**Figure 2.**
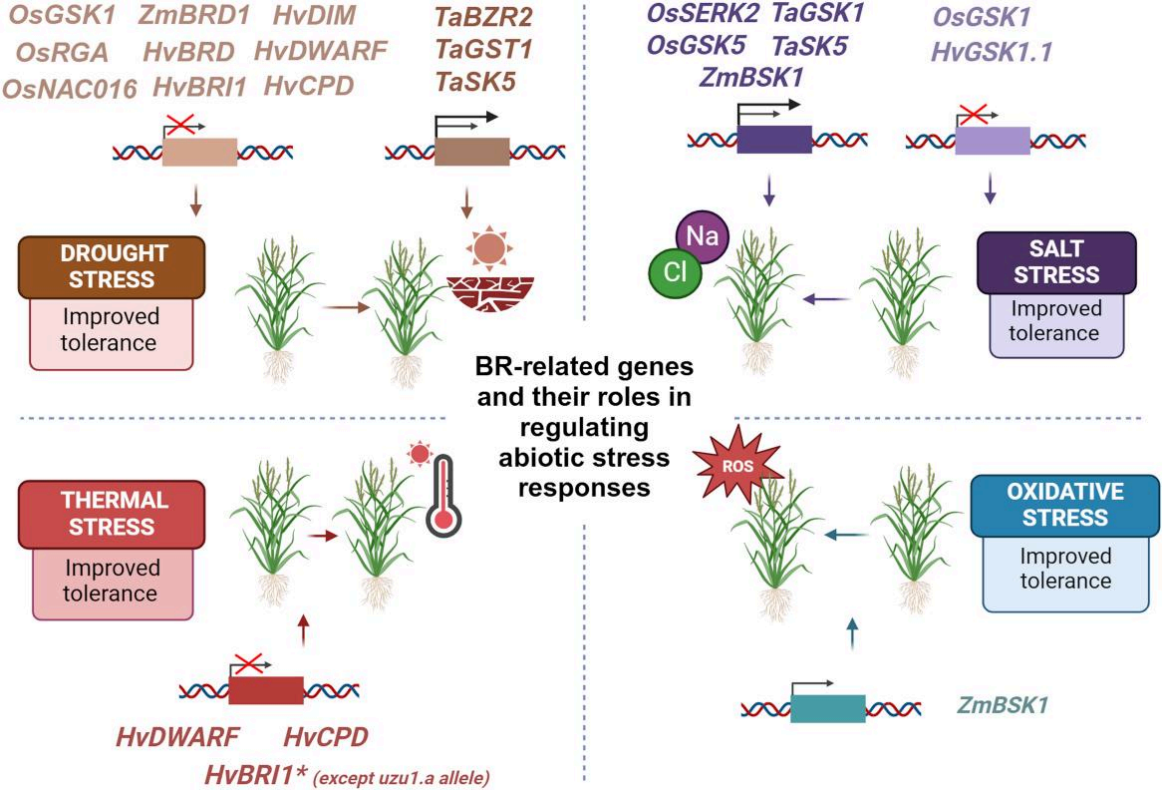
Effects of manipulating BR biosynthesis genes and genes encoding positive and negative regulators of BR signaling in rice, maize, wheat, and barley on improved tolerance of these crop species to drought, salinity, thermal, and oxidative stress. Full names of the cereal genes are given in the text. Created with BioRender.com. Os, *O. sativa*; Zm, *Z. mays*; BRD1, brassinosteroid-deficient dwarf1; Hv, *H. vulgare*; DIM, DIMINUTO; RGA, α-subunit of the rice heterotrimeric G protein; BRD, brassinosteroid-6-oxidase; *NAC016*, gene from the NAM, ATAF, and CUC family; BRI1, brassinosteroid-insensitive1; CPD, constitutive photomorphogenic dwarf; Ta, *T. aestivum*; BZR, brassinazole resistant; GST1, glutathione S-transferase; SK, SHAGGY-like kinase; SERK, somatic embryogenesis receptor-like kinase; BSK, brassinosteroid signaling kinase; Na, sodium; Cl, chloride; ROS, reactive oxygen species.

However, it should be noted that the involvement of BR signaling components in regulating the drought response might be species-dependent or depend on specific components of the pathway (Fig. 2). For instance, wheat plants overexpressing the *brassinazole-resistant2* (*TaBZR2*) gene showed increased drought tolerance, whereas RNAi-dependent downregulation of this gene led to reduced drought tolerance. A molecular mechanism of this phenomenon was proposed: the TaBZR2 transcription factor directly binds to the promoter of the *glutathione S-transferase1* (*TaGST1*) gene to stimulate its expression (Fig. 2). TaGST1 actively scavenges drought-induced reactive oxygen species (ROS), namely superoxide anions (O_2_^−^). Thus, TaBZR2 positively regulates drought responses by activating TaGST1 and participates in the interplay between the BR and drought signaling pathways (Cui et al. 2019).

Intriguing results were obtained in a recent study in which BRI1-EMS-SUPPRESSOR1 (BES1) transcription factor family members were expressed in wheat under various stress conditions, including drought. Most *TaBES1*-like genes were downregulated under drought conditions. Furthermore, the gene expression profiles were compared between drought-tolerant and drought-sensitive wheat cultivars grown under drought conditions. Interestingly, the expression levels of some *TaBES1* genes (*TaBES1-3B2* and *TaBES1-3D2*) were elevated in drought-tolerant cultivars but significantly repressed in drought-sensitive cultivars. However, the *TaBES1-6D* gene displayed the opposite expression pattern (Wang et al. 2023a). Perhaps even within one gene family encoding components of the BR signaling pathway (in the same species), some genes may show different (or even opposite) expression patterns during plant reactions to the same stress factor.

A similar type of differential expression was reported among genes belonging to the *GSK* family in rice and Arabidopsis (*Arabidopsis thaliana*) during the reactions of these species to various abiotic stress conditions, including drought (Ahmar et al. 2023b). In Indian dwarf wheat (*Triticum sphaerococcum*), 2 missense (gain-of-function) mutations in the highly conserved TREE (Thr-Arg-Glu-Glu) motif of GSK family members, which undergoes several rounds of phosphorylation/dephosphorylation during the regulation of stability of these kinases (Li and Nam 2002; Li et al. 2021), led to improved drought tolerance and elevated phosphate and nitrogen accumulation. These physiological responses might represent adaptations to the arid climates of some parts of India and Pakistan (Gupta et al. 2021; Zolkiewicz and Gruszka 2022). Importantly, Indian dwarf wheat, which is characterized by short stature, erectness, dense spikes, and small, spherical grains, was a staple crop in India and Pakistan from the Bronze Age until the 20th century (Gupta et al. 2021). More recently, gain-of-function mutations in the TREE motif of TaGSK3 were shown to enhance drought tolerance in wheat (Guo et al. 2024).

Notably, most studies in cereal species were focused on the effects of drought only on vegetative development. However, the transition from the vegetative to reproductive phase of development is the most sensitive stage, as any stress encountered during this period severely affects plant physiology and yield (Gupta et al. 2020; Cohen et al. 2021). The mechanisms underlying plant adaptation to drought stress during this key transition phase remain largely unknown and require further study (Templer et al. 2017). Our knowledge about the role of BRs in regulating reproductive development under drought conditions is still very limited, even in the model crop species rice (Zhang et al. 2019b; Alqudah et al. 2021). Therefore, since reproductive development and yield are crucial aspects of cereal crop biology, it is of upmost importance to characterize the BR-dependent molecular mechanisms regulating the responses to drought exerted at this developmental stage in rice and other cereals, particularly in the face of global climate change. Nevertheless, research conducted in the above-mentioned cereal species has led to the selection of genes involved in BR biosynthesis or signaling that could become targets of genetic or biotechnological modification aimed at improving drought tolerance. However, it should also be kept in mind that some members of gene families (particularly involved in the BR signaling) may show different (or even opposite) expression patterns during plant responses to drought.

### Reactions of cereal BR mutants to salinity stress

Salinity is one of the major environmental stresses that negatively affect plant growth, development, and the yield and quality of cereal crops. Therefore, in order to improve the tolerance of cereals to salinity stress, several attempts have been made involving the genetic or biotechnological manipulation of BR-related genes. However, it should be kept in mind that research on the roles of individual components of the BR signaling pathway in the salt stress response in cereals led to diverse conclusions (as described below). Overexpressing the *somatic embryogenesis receptor-like kinase2* (*OsSERK2*) gene encoding a BR receptor component increased tolerance to salt stress in rice (Dong et al. 2020). Overexpressing the *brassinosteroid signaling kinase1* (*ZmBSK1*) gene in maize also improved salt stress tolerance (Liu et al. 2022a) (Fig. 2). Further study indicated that another gene of the *BSK* family in maize, *ZmBSK7*, is rapidly induced by the salt stress, and *ZmBSK7* overexpression enhances salt tolerance. ZmBSK7 stimulates the activities of antioxidant enzymes, diminishes plasma membrane damage, promotes K^+^ accumulation, and reduces the Na^+^/K^+^ ratio (Zhang et al. 2024a). Both *OsSERK2* and *ZmBSK1* encode positive regulators of BR signaling in these cereal species. Interestingly, the above-mentioned loss-of-function mutation of *OsGSK1* (encoding a negative regulator of BR signaling) in rice led to the development of mutant plants with improved tolerance to various abiotic stresses, including salinity (Koh et al. 2007). More recent studies in barley indicated that mutant lines developed through CRISPR/Cas9-mediated knockout or RNAi-mediated silencing of *HvGSK1.1* also showed improved tolerance to salt stress (Kloc et al. 2020, 2024) (Fig. 2). These phenotypic traits might have resulted from enhanced BR signaling and the activity of HvGSK1.1 as a negative regulator of salt tolerance in barley. Based on these results, the *HvGSK1.1* gene could represent a target of genetic editing aimed at developing barley plants with improved agronomic traits (Kloc et al. 2024).

Notably, studies on the roles of plant GSK proteins in the salt stress response led to the conclusion that different members of the GSK3 family regulate this response in different (sometimes even opposite) manners in various cereal species. For instance, the *OsGSK5/SHAGGY-like kinase41* (*OsSK41*) gene in rice (Thitisaksakul et al. 2017) and the *TaGSK1* and *TaSK5* genes in wheat (He et al. 2012; Christov et al. 2014) positively regulate salinity tolerance (Fig. 2). In the case of rice, this salinity stress tolerance was related to carbohydrate metabolism and was achieved, at least partially, through preferential carbon allocation to starch in roots (Thitisaksakul et al. 2017). BR signaling and carbohydrate metabolism are interconnected and regulate plant growth in the light-/darkness-dependent manner. Under light conditions, carbohydrates repress BR signaling and plant growth. Although this inhibitory effect is independent of the carbohydrate signaling pathway mediated by the target of rapamycin protein (the pathway has not been fully elucidated), it requires the GSK protein, which is stabilized by carbohydrate signaling in the light. On the contrary, carbohydrate signaling stimulates BR signaling and plant growth in the dark. The light-/darkness-dependent interplay between carbohydrate metabolism and BR signaling allows for the optimal adjustment of plant growth to environmental conditions (Zhang et al. 2021; Zolkiewicz and Gruszka 2022). Interestingly, *TaSK5* overexpression also improved drought tolerance (Fig. 2). However, it should be kept in mind that the functions of these wheat *GSK* genes in salt stress tolerance were inferred in these studies based on heterologous expression in *Arabidopsis* (He et al. 2012; Christov et al. 2014). In an earlier study, *TaGSK1* in wheat was shown to be induced by salinity stress, and the expression of this gene was stronger in salt-resistant genotypes than in salt-sensitive ones. These results led to the conclusion that *TaGSK1* plays a positive role in regulating salinity stress tolerance in this cereal species (Chen et al. 2003) (Fig. 2).

Based on these findings, overexpressing positive regulators of BR signaling, including OsSERK2 in rice and ZmBSK1 and ZmBSK7 in maize, improved salt stress tolerance in these species. Different GSK3 proteins are thought to regulate these responses in different (sometimes opposite) manners in cereal species. However, knockout or RNAi-mediated silencing of *HvGSK1.1* in barley also improved tolerance to salt stress. These results suggest that the above-mentioned genes could be used as the targets of genetic and biotechnological manipulation aimed at developing cereals with improved salt stress tolerance.

### Reactions of cereal BR mutants to thermal stress

Global climate change has brought about the increased occurrence of heat stress, which has become a limiting factor of plant growth and development and, consequently, agricultural production (Zhang et al. 2022; Cao et al. 2024). Studies on the roles of BRs in regulating the growth, development, and yield of cereals in response to thermal stress have been conducted in several species—rice, wheat, and barley. However, different aspects of this process and different genes involved in BR biosynthesis, BR decomposition, and the positive and negative regulation of BR signaling were analyzed in these species.

High temperature (40 °C) negatively affects panicle growth and spikelet formation in rice; rice spikelet development is dependent on BRs. Analyses of the expression profiles of genes involved in BR biosynthesis and decomposition indicated that high temperature induces BR decomposition, leading to decreased BR accumulation. BRs also promote the transport of sucrose from leaves to developing panicles and sucrose utilization under optimal and high-temperature conditions (Chen et al. 2021). Importantly, high temperature significantly impaired both these processes (Zhang et al. 2018a; Chen et al. 2021). Therefore, high temperatures are thought to inhibit spikelet development through decreased sucrose transport and utilization evoked by stress-induced BR decomposition (Chen et al. 2021).

The above-mentioned gain-of-function mutation in the TREE motif of TaGSK3 improved heat tolerance in wheat. The mutant plants maintained higher relative water content under heat stress conditions due to reduced stomatal-mediated water loss. The improved heat tolerance was achieved in the mutant through a reduced rate of cell membrane damage and decreased accumulation of ROS (Guo et al. 2024). Another study in Indian dwarf wheat indicated that an amino acid substitution in the TREE motif of TaGSK3 improved heat tolerance by enhancing the phosphorylation and stability of the target phytochrome-interacting factor4 (TaPIF4) transcription factor under heat stress condition (Cao et al. 2024). Notably, the PIF4 transcription factor interacts with the BES and brassinazole-resistant (BZR) transcription factors; this interaction helps integrate BR and environmental responses (Oh et al. 2012; Nolan et al. 2017).

In barley, interesting conclusions were reached upon analysis of the reaction of semi-dwarf BR mutants to high temperatures. In this experiment, the above-mentioned Bowman NILs, representing recently identified mutants defective in the function of the BR receptor HvBRI1 or the BR biosynthesis enzymes HvDIM, HvCPD, and HvBRD, were cultivated under optimal (control) and heat stress conditions and compared with the reference cultivar “Bowman” (Dockter et al. 2014). The group of genotypes included the original BR-insensitive *uzu1.a* mutant, which has been the source of semi-dwarfism in barley breeding in Northeast Asia (Chono et al. 2003; Saisho et al. 2004). Comparative analysis indicated that at the elevated temperature, the *uzu1.a* mutant showed extreme dwarfism in contrast to the other (recently identified) BR mutants, which retained their semi-dwarf stature under stress conditions. The *uzu1.a* mutant is exceptional in terms of its temperature-dependent phenotype, even compared with allelic mutants carrying mutations in the same BR receptor gene (*HvBRI1*) (Fig. 2). This could help explain why barley cultivars into which the *uzu1.a* allele had been introduced during previous breeding programs did not spread globally (particularly from Japan, South Korea, and the coastal region of China into the interior of Asia) during the Green Revolution. Taking into account that the temperature-sensitive phenotype is specific only to the *uzu1.a* mutant, the recently identified mutants may constitute alternatives for “*uzu*” barley in future breeding programs, especially in the face of ongoing climate change (Dockter et al. 2014).

A subsequent study examined whether the improved tolerance to high temperatures displayed by the majority of semi-dwarf barley BR mutants is dependent on their genetic background. In this study, semi-dwarf mutants defective in BR perception (mutation in the *HvBRI1* gene) or BR biosynthesis (mutations in the *HvCPD* and *HvDWARF* genes), derived from 2 different cultivars, were compared with their respective reference cultivars during growth and development under optimal (control) and heat stress conditions. Importantly, all the semi-dwarf BR mutants showed improved tolerance to high temperatures compared with their respective reference cultivars (Sadura et al. 2019). These results indicate that increased tolerance to high temperatures is a common feature of semi-dwarf barley BR mutants (except for the above-mentioned original BR-insensitive *uzu1.a* mutant), independent of their genetic background (Fig. 2), confirming that the recently identified mutants could serve as alternatives in future barley breeding programs (Dockter et al. 2014; Sadura et al. 2019). However, it should be kept in mind that the molecular mechanism responsible for the increased tolerance of the semi-dwarf, barley BR mutants to high temperatures remains largely unknown, and further studies, particularly at the transcriptomic and metabolic levels, are needed to fully clarify this phenomenon.

Importantly, in the same study, the reactions of the semi-dwarf BR barley mutants to low temperature (−6 or −8 °C) were analyzed. Two of the mutants, one deficient in BR biosynthesis and the other defective in BR perception, were less tolerant to frost than their respective reference cultivar, whereas another BR-deficient mutant (of a different background) showed similar frost tolerance to its reference cultivar. These results suggest that a relationship may exist between frost tolerance and the levels of BR and ABA accumulation in barley (Sadura et al. 2019). However, the effect of the genetic background on this stress response should not be excluded, and further research on this topic is needed.

In conclusion, a group of BR-related genes involved in BR biosynthesis, as well as positive and negative regulators of BR signaling, may be regarded as candidates for genetic manipulation aimed at improving tolerance to high temperatures (particularly in wheat and barley). The application of new BR-related cereal germplasm with improved heat tolerance may be particularly beneficial in the face of global climate change to maintain or expand the area of cereal cultivation. However, further research (particularly at the transcriptomic and metabolic levels) is needed to fully elucidate the molecular mechanism of the BR-dependent regulation of the responses of cereals to thermal stress.

### Insight into BR-dependent mechanisms of plant reactions to oxidative stress

Plants exposed to environmental stress must coordinate various processes, including biochemical adjustments, in order to adapt to stress conditions (Rao and Chaitanya 2016). Disturbances in metabolic activity during exposure to stress cause the overproduction of ROS. Plants need to maintain redox homeostasis to counteract the detrimental effects of excessive ROS accumulation, which may lead to oxidative stress (Noctor et al. 2014; Choudhury et al. 2016). Oxidative stress poses a major constraint to crop productivity (Mittler 2002). Disturbances in BR biosynthesis or BR signaling affect the accumulation of non-enzymatic antioxidants in barley (Gruszka et al. 2018). BRs rapidly and directly stimulate the activity of ZmBSK1 in maize, which positively regulates tolerance to this stress (Fig. 2). Interestingly, BRs do not influence accumulation of ZmBSK1 (the protein activity is stimulated through posttranslational modification). A study using *ZmBSK1*-overexpressing lines and *ZmBSK1*-knockdown lines demonstrated that ZmBSK1 is required for the BR-stimulated production of H_2_O_2_ (Liu et al. 2022b). BRs stimulate the activity of the nicotinamide adenine dinucleotide phosphate (NADPH) oxidase, which leads to increased apoplastic H_2_O_2_ accumulation and the regulation of plant development and stress responses (Xia et al. 2015b). The BR-induced accumulation of H_2_O_2_ stimulates ABA biosynthesis. In turn, the increase in ABA biosynthesis causes prolonged H_2_O_2_ accumulation and the induction of stress tolerance (Zhou et al. 2014). Importantly, the ZmBSK1 kinase phosphorylates the calcium/calmodulin-dependent protein kinase (ZmCCaMK), and this BR-stimulated protein interaction plays an important role in positively regulating the drought tolerance of maize plants (Liu et al. 2021). Moreover, this ZmBSK1-mediated phosphorylation of the ZmCCaMK kinase is required for the BR-dependent stimulation of the NADPH oxidase to modulate the accumulation of H_2_O_2_, a secondary messenger molecule that participates in BR signaling and stress responses. The ZmBSK1–ZmCCaMK interaction is important for enhancing plant tolerance to oxidative stress through the stimulation of antioxidant defense and diminishing electrolyte leakage (Liu et al. 2022b).

Therefore, although preliminary insight has been gained into the role of BRs in regulating the responses of cereals to oxidative stress, and some candidate genes for genetic or biochemical modification have been selected, several aspects of this process need to be elucidated. For example, which molecular mechanisms mediate the BR-dependent regulation of the oxidative stress response at the transcriptional, metabolic, or epigenetic level? If and how are these mechanisms regulated by interhormonal crosstalk? These questions need to be answered to fully clarify the BR-dependent regulation of the oxidative stress response in cereals and to develop methods for its improvement.

## Mortal combat—manipulating BR homeostasis enhances defense against biotic stress in cereals

**Figure 3.**
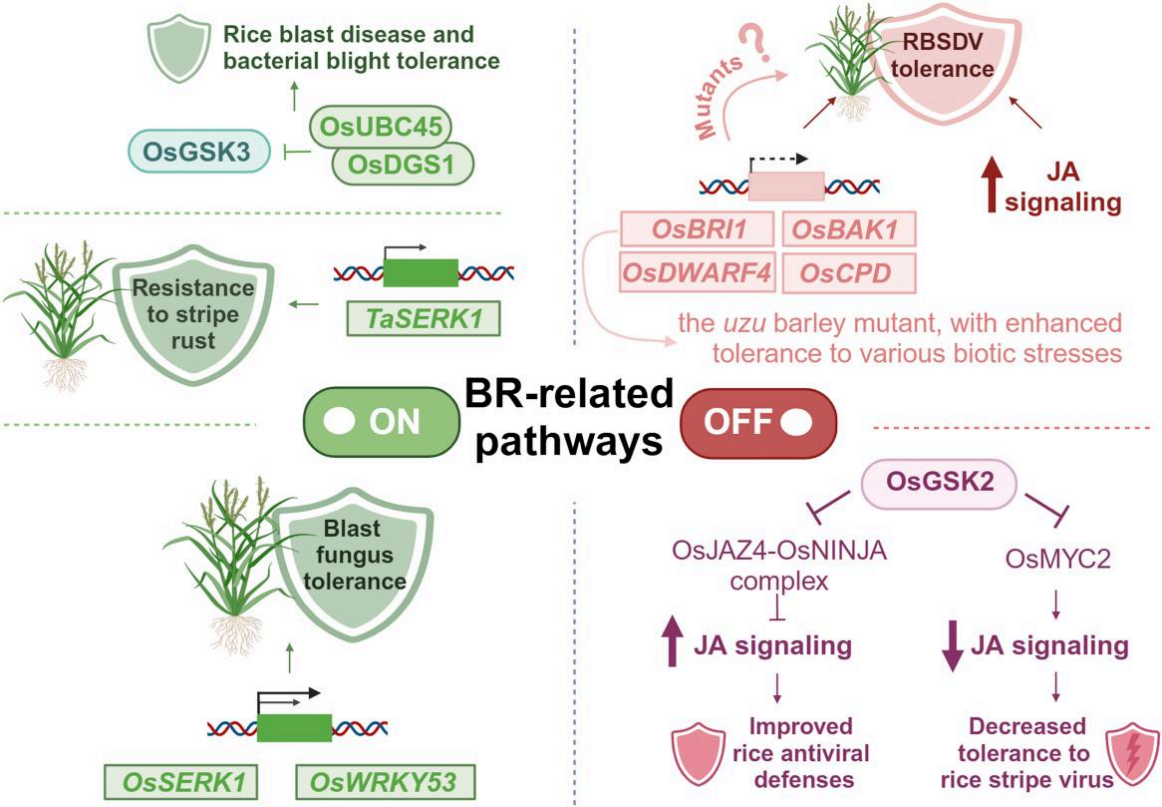
Effects of manipulating BR biosynthesis and BR signaling genes (positive and negative regulators of this process) in rice, wheat, and barley on the tolerance of these crop species to various biotic stresses. Full names of the cereal genes are given in the text. Created with BioRender.com. Os, *O. sativa*; UBC, ubiquitin-conjugating enzyme; DGS1, decreased grain size1; Ta, *T. aestivum*; BAK1, BRI1-associated receptor kinase1; CPD, constitutive photomorphogenic dwarf; WRKY53, transcription factor belonging to the WRKY (Trp-Arg-Lys-Tyr domain) family; JAZ4, jasmonate ZIM-domain4; MYC2, transcription factor belonging to the Myelocytomatosis family.

Genetic manipulation of BR signaling components in cereals using biotechnological approaches may result in enhanced tolerance to biotic stress (Choudhary et al. 2012). Constitutive overexpression of the rice *OsSERK1* gene, which encodes a component of the BR receptor complex, increased the tolerance of transgenic rice to blast fungus (Hu et al. 2005) (Fig. 3). On the other hand, BRs induce the hypersensitivity of rice plants to some viruses, including the rice black-streaked dwarf virus (RBSDV), which leads to deleterious disease symptoms (He et al. 2017). Interestingly, analysis of gene expression profiles in rice plants during their response to RBSDV indicated that the expression of the BR biosynthetic genes *OsDWARF4* and *OsCPD* and the BR signaling-related genes *OsBRI1* and *BRI1-associated receptor kinase1* (*OsBAK1*) was significantly reduced, in contrast to jasmonic acid (JA) signaling-related genes, which are upregulated during plant responses to viral infection (Fig. 3). Therefore, the JA-mediated increased tolerance of rice plants to RBSDV infection involves decreased expression of BR-related genes (He et al. 2017). These findings indicate that rice mutants defective in BR biosynthesis or signaling (or showing decreased expression of BR-related genes) may display increased tolerance to RBSDV infection (Fig. 3). The different effects of the genetic manipulation of BR-related genes on tolerance to fungal and viral infections may stem from the fact that pathogens may activate different pathogen response signaling pathways. Plants perceive pathogen-associated molecular patterns (PAMP) through the activation of pathogen recognition receptors, which results in the initiation of PAMP-triggered immunity (PTI) (Jones and Dangl 2006; Schwessinger and Zipfel 2008). Additionally, pathogen-derived effectors may be perceived by resistance (R) proteins, thereby initiating effector-triggered immunity, which results in strong defense responses, constituting a form of programmed cell death (Yang et al. 2011). The above-mentioned somatic embryogenesis receptor-like kinase (SERK) coreceptors involved in BR perception form a convergence point between BR signaling and PTI (Vert 2008; Choudhary et al. 2012).

GSK3 proteins also regulate the responses of cereals to viral infection. GSK3s undergo various mechanisms to regulate the stability and activity of their target proteins. In addition to functioning as major regulators of BR signaling, these proteins function as multifaceted hubs of various signaling pathways and modulators of development, reproduction, and responses to diverse environmental conditions, including biotic stress. The diverse effects of GSK3 activity result from their broad range of target proteins, allowing them to participate in various response pathways (Zolkiewicz and Gruszka 2022). In rice, the GSK protein OsGSK2 regulates this process by interacting with a transcription factor that participates in the JA signaling pathway. OsGSK2 phosphorylates the jasmonate ZIM-domain4 (OsJAZ4) transcription factor to inhibit its association with another transcription regulator, OsJAZ11, and the formation of the OsJAZ4–OsNINJA corepressor complex (Fig. 3). The formation of the OsJAZ4–OsJAZ11 complex facilitates the proteasomal degradation of OsJAZ4. This interaction leads to the degradation of OsJAZ4, which negatively regulates JA signaling and antiviral responses. Thus, OsGSK2 enhances antiviral defenses in rice by activating JA signaling by interacting with, phosphorylating, and destabilizing OsJAZ4 (He et al. 2020) (Fig. 3).

On the other hand, the same rice GSK protein, OsGSK2, phosphorylates the myelocytomatosis2 (OsMYC2) transcription factor, a positive regulator of the JA signaling pathway (Uji et al. 2016). Interestingly, this OsGSK2-mediated phosphorylation leads to the degradation of OsMYC2, thereby reducing JA-dependent tolerance to rice stripe virus (Fig. 3). Moreover, using activation-tagged T-DNA insertion, the rice *slender grain Dominant* (*slg-D*) mutant was identified. This mutant is characterized by the increased accumulation of endogenous BR and improved resistance against rice stripe virus (Hu et al. 2020). Therefore, OsGSK2-mediated phosphorylation may affect both negative and positive regulators of JA signaling (Fig. 3). This dual regulation is thought to fine-tune the responses of rice to viral infection (Zolkiewicz and Gruszka 2022).

The rice gene *OsUBC45*, also known as *small grain3* (*SMG3*), encodes a ubiquitin-conjugating enzyme that participates in endoplasmic reticulum-associated protein degradation. OsUBC45 interacts with and promotes the degradation of the OsGSK3 kinase. Consequently, OsUBC45 stimulates BR signaling and contributes to increased plant yield and tolerance against rice blast disease and bacterial blight (Fig. 3). This increased plant tolerance is achieved via amplification of the above-mentioned PTI response (Wang et al. 2023b; Yu and Xie 2024). The *decreased grain size1* (*OsDGS1*) gene encodes a RING-type E3 ligase that interacts with OsUBC45 to facilitate the ubiquitin-dependent degradation of OsGSK3 and the aquaporin plasma membrane intrinsic protein2;1 (OsPIP2;1), thereby influencing rice yield and immunity (Wang et al. 2024) (Fig. 3). Notably, both the OsGSK3 kinase and the OsPIP2;1 aquaporin are negative regulators of PTI (Gao et al. 2019; Wang et al. 2023b). Importantly, *OsDGS1* has positive effects on grain size, grain number, and 1,000-grain weight (Zhu et al. 2021; Li et al. 2023). In addition, analysis of *OsDGS1* overexpression lines suggested that this gene stimulates plant immunity and rice blast resistance by enhancing ROS accumulation, activating the mitogen-activated protein kinase (MAPK) signaling pathway and increasing the expression of defense-related genes. Overexpressing *OsDGS1* stimulates the immunity response and positively influences rice yield. This genetic manipulation is a good example of how to achieve the balance between enhanced stress tolerance and the maintenance (improvement) of yield. Thus, *OsUBC45-DGS1* represents a potential target of genetic manipulation for rice improvement through breeding (Wang et al. 2024).

BRs negatively regulate the GA-mediated and salicylic acid (SA)–mediated immunity of rice roots against the oomycete *Pythium graminicola*. Interestingly, this pathogen utilizes BRs as virulence factors and exploits BR signaling to inflict disease through the negative, immune-suppressive effect of BRs on the GA and SA signaling pathways. In fact, the pathogen-mediated manipulation of BR homeostasis is a major virulence strategy (De Vleesschauwer et al. 2012), suggesting that producing defects in BR biosynthesis or BR signaling represents an alternative strategy for developing new rice cultivars with improved tolerance to this pathogen.

The expression of defense-related genes in rice may also be regulated by OsWRKY53 (Chujo et al. 2007), a transcription factor that positively regulates BR signaling (Tian et al. 2017). Proteins belonging to the WRKY (Trp-Arg-Lys-Tyr domain) family play various roles in plant responses to biotic and abiotic stress by modulating target gene expression (Rushton et al. 2010). Apart from regulating plant architecture (in a BR-dependent manner), OsWRKY53 positively regulates pathogen defense responses as well. *OsWRKY53* expression is induced by the chitin oligosaccharide elicitor and by infection with the blast fungus *Magnaporthe grisea*. Defense-related genes, including pathogenesis-related (PR) genes, were upregulated in rice plants overexpressing *OsWRKY53*, suggesting that OsWRKY53 is involved in basal defense responses against pathogen infection. Overexpressing *OsWRKY53* enhanced resistance to the blast fungus *M. grisea* (Fig. 3). Moreover, the morphology of transgenic plants overexpressing *OsWRKY53* was similar to that of the wild type. These findings indicate that OsWRKY53 positively regulates both the BR signaling and defense signaling pathways (Chujo et al. 2007). Therefore, overexpressing *OsWRKY53* might represent another strategy for achieving the balance between the maintenance of plant growth (development) and enhanced resistance to this pathogen.

Many leucine-rich repeat receptor-like kinases (including SERK proteins) play crucial roles in regulating defense responses (Brandt and Hothorn 2016). The extracellular domain of SERK proteins perceives microbe-derived or host-derived molecular patterns and activates the kinase domain of the SERK receptor to induce innate immune responses (Liu et al. 2020). The rice OsSERK2 kinase positively regulates plant immunity to *Xanthomonas oryzae* pv. *oryzae* (Chen et al. 2014b). In wheat, the TaSERK1 kinase positively regulates high temperature–dependent resistance against stripe rust caused by the fungal pathogen *Puccinia striiformis f. sp. tritici* (Shi et al. 2023) (Fig. 3). *TaSERK1* gene expression increases in response to inoculation under high-temperature conditions. Using virus-induced gene silencing, the authors concluded that TaSERK1 positively regulates high temperature–dependent plant resistance to stripe rust in wheat at the seedling stage (as *TaSERK1*-silenced plants showed decreased defense responses). TaSERK1 kinase interacts and phosphorylates the chaperone protein DnaJA7 (TaDJA7) belonging to the heat shock protein family. TaDJA7 is a positive regulator of wheat resistance to stripe rust, as silencing *TaDJA7* decreased defense responses. Phosphorylated TaDJA7 is thought to regulate the expression of *PR* genes to stimulate immune responses. These findings indicate that stripe rust infection is perceived by TaSERK1 (under high-temperature conditions), and this kinase initiates signaling to activate TaDJA7, thereby activating temperature-dependent plant resistance (Shi et al. 2023). The dependence between temperature and plant defense responses is an interesting phenomenon; it was recently suggested that temperature variations have substantial effects on plant defense mechanisms, including *R* gene-mediated resistance (Cohen and Leach 2020).

A similar conclusion came from a recent study by Rys et al. (2024), who reported that heat pretreatment induced shifts in plant–pathogen (powdery mildew) interactions toward higher susceptibility in barley. In this study, 2 mutants with disturbances in BR biosynthesis or perception had slightly higher resistance to powdery mildew (Bgt, powdery mildew of wheat) than their reference cultivar (at 20 °C), and one of the BR-deficient mutants showed substantially higher resistance to *Blumeria hordei* (at 20 °C and after heat stress). These findings suggest that BRs play a role in the temperature-dependent pathogen response in barley. Moreover, one of the BR-deficient mutants of the *HvDWARF* gene maintained high resistance to barley powdery mildew race A6 despite heat pretreatment. The higher resistance of the BR-deficient mutant to barley powdery mildew, even after high-temperature stress, may constitute a promising alternative from a practical standpoint for future breeding programs. However, in this case, an allele-specific effect and dependence on the genetic background of the analyzed mutants and cultivars should also be taken into account (Rys et al. 2024).

The mutation in the barley *uzu* mutant was shown to improve resistance against several pathogens: leaf blast disease (caused by *M. grisea*), take-all of roots (caused by *Gaeumannomyces graminis* var. *tritici*), eyespot disease of stems (caused by *Oculimacula* spp.), and crown rot disease of stems (caused by *Fusarium* fungi) (Goddard et al. 2014; Chen et al. 2014a). Subsequently, the *uzu* mutation was shown to confer broad spectrum, substantially improved resistance against several pathogens: barley stripe mosaic virus, the necrotrophic net blotch pathogen *Pyrenophora teres*, and the hemibiotrophic fungus *Fusarium culmorum* that causes Fusarium head blight (Ali et al. 2014) (Fig. 3). However, the disease resistance of the *uzu* mutant lines is environment dependent. The *uzu* lines display enhanced resistance to pathogens with a short biotrophic phase or a necrotrophic lifestyle, but not to the biotroph *Blumeria graminis* or to the long asymptomatic phase-type leaf pathogen *Ramularia collo-cygni* (Goddard et al. 2014).

In conclusion, the genetic manipulation of several BR-related genes has already been successfully applied (particularly in rice) to improve the immunity response and resistance to pathogens. Importantly, such genetic modifications helped to achieve the balance between enhanced tolerance to stress and the maintenance (improvement) of plant yield or development. However, further research is needed to expand the list of BR-related genes that could become targets of genetic modification in rice and other cereal species. Some promising results were obtained in barley in terms of improved resistance of the BR-deficient mutant to powdery mildew, which was maintained after exposure to high-temperature stress, representing an alternative for future breeding programs. However, further research is needed to shed light on the molecular mechanism underlying this phenomenon.

## Interplay between microRNAs and BRs in regulating stress responses in cereals

miRNAs are small (ranging from 20 to 24 nucleotides in length), endogenous RNAs that function in posttranscriptional gene regulation by guiding their target mRNAs for degradation and/or through translational repression (Li and Zhang 2016). Thus, miRNAs play an indirect but crucial role in plant development and are important regulators of genes involved in plant responses to biotic and abiotic stress (Sun et al. 2019; Li et al. 2020; Begum 2022; Zhang et al. 2022). Most of miRNAs are highly conserved among various plant species, including cereal crops (Zhang et al. 2022). Moreover, miRNAs constitute hubs of complicated gene regulatory networks (Raza et al. 2023). However, the miRNA-mediated networks that regulate plant development and stress responses are still not fully elucidated: the functions of many miRNAs are still unclear, and consequently, their coordinated action is even more poorly understood (Cui et al. 2014; Liu et al. 2018; Raza et al. 2023). Therefore, miRNAs that play pivotal roles in plant stress tolerance and adaptation may have significant potential for agronomic trait improvement in crop species through genetic engineering (Budak and Zhang 2017; Zhang et al. 2022; Mondal et al. 2023).

Numerous miRNA gene promoters contain *cis*-acting regulatory elements related to stress responses, indicating that plants respond to environmental stress by modulating miRNA expression (Dong et al. 2022). In response to abiotic stress conditions, diverse miRNAs are differentially expressed as part of an adaptive strategy and modulate the expression of genes involved in stress responses (Li et al. 2020; Singroha et al. 2021). A group of miRNAs that participate in the responses of model and crop species to various abiotic stresses was recently assembled (Mondal et al. 2023). For instance, in rice seedlings subjected to drought stress, 16 miRNAs were upregulated, whereas 17 miRNAs were downregulated (Zhang et al. 2016). In maize leaves, 8 miRNAs were upregulated, while 13 miRNAs were downregulated during the drought response (Liu et al. 2019).

Some miRNAs regulate the expression of genes involved in phytohormone biosynthesis or signaling (Li et al. 2020; Dong et al. 2022). Moreover, a subgroup of these miRNAs participates in several biosynthesis or signaling pathways of various phytohormones and therefore constitutes another point of interhormonal crosstalk. Phytohormones also regulate the accumulation of miRNAs. This mutual interplay between miRNAs and phytohormones allows plants to efficiently adapt to constantly changing environmental conditions (Li et al. 2020). Notably, miRNAs are important for the precise regulation of gene expression in a tissue-specific manner, particularly compared with the plant-wide actions of phytohormones (Dong et al. 2022).

**Figure 4.**
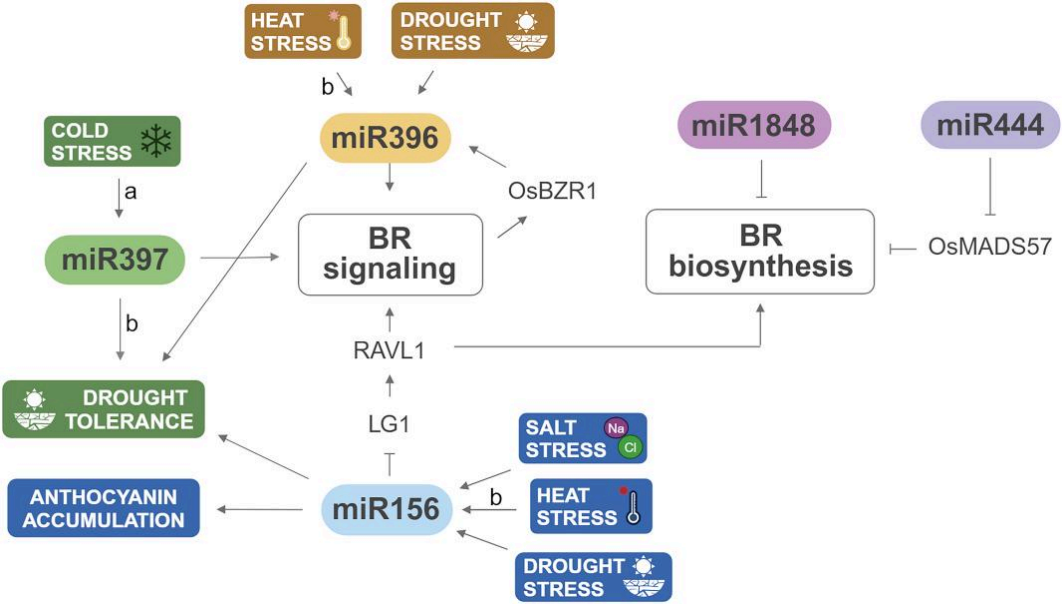
Mechanisms of the interplay between miRNAs and BR biosynthesis and BR signaling genes during the responses of monocots to various environmental stresses. Most of the genetic mechanisms were described in rice. Mechanisms described in other monocots (*B. distachyon* and maize) are indicated with the letters “a” and “b,” respectively. Full names of the genes are given in the text. Created with BioRender.com. miR, microRNA; Os, *O. sativa*; BZR, brassinazole-resistant; MADS57, gene belonging to the MADS-box family.

Currently, relatively little is known about the BR-dependent regulation of miRNA expression or the miRNA-mediated regulation of BR-related gene expression, even in the model cereal crop rice (Li et al. 2020). However, some initial insights into the miRNA-BR dependencies were recently gained; for instance, some miRNAs influence BR biosynthesis and signaling in rice (Li et al. 2020). A transposable element-derived miRNA downregulates the BR biosynthesis gene *BR-6-oxidase* (*OsBR6ox*) and diminishes BR accumulation in rice (Wei et al. 2014). However, the functions of only 2 miRNAs in the miRNA-mediated regulation of BR biosynthesis in rice have been characterized in detail. Interestingly, these miRNAs play opposite roles in regulating BR biosynthesis. miR1848 targets the transcript of *Cytochrome P51G3* (*OsCYP51G3*), a gene encoding obtusifoliol 14α-demethylase, which catalyzes an early step in sterol/BR biosynthesis. Interestingly, miR1848 overexpression leads to decreased BR accumulation and influences plant responses to salt stress (Xia et al. 2015a). On the other hand, miR444 positively regulates the expression of BR biosynthesis genes in rice. This positive impact on BR biosynthesis is mediated by the miR444-mediated silencing of MCM-AGAMOUS-DEFICIENS-SRF (MADS)-box gene *OsMADS57*, which encodes a repressor of BR biosynthesis genes in rice (Jiao et al. 2020). These findings suggest that miR1848 directly regulates BR biosynthesis, whereas miR444 has an indirect, positive impact on BR biosynthesis (Fig. 4).

miR396 positively regulates BR signaling in rice, and the miR396 expression is directly enhanced by the key transcription factor OsBZR1, which regulates BR-dependent gene expression. In plants overexpressing miR396, BR signaling was stimulated, which resulted in the elevated expression of miR396, pointing to a positive feedback loop (Tang et al. 2018). Importantly, miR396 plays a significant role in abiotic stress responses (Zhang et al. 2022). miR396 expression is induced by drought, and its overexpression stimulates drought tolerance (Li et al. 2017). Thus, the positive feedback loop between miR396 and BR signaling, the drought-induced expression of miR396, and the stimulation of drought tolerance by miR396 might make the interplay between miR396 and BR signaling another promising target of further genetic manipulation in rice and other cereal crops, particularly in the face of global climate change (Fig. 4).

BRs negatively regulate the miRNA-mediated translation inhibition of target genes by influencing distribution of AGO1 proteins, which play crucial roles in miRNA-mediated gene silencing in the endoplasmic reticulum (Wang et al. 2021). The interplay between miRNAs and BRs might also be influenced by environmental conditions. In the monocot model species *Brachypodium distachyon*, miR397 is substantially upregulated (∼15-fold) in response to cold stress (Zhang et al. 2009). Importantly, miR397 is directly involved in regulating the BR response and yield in rice. Overexpressing miR397 resulted in a significant increase in BR sensitivity and grain yield (Zhang et al. 2013) (Fig. 4). Therefore, it is important to verify whether the cold stress–induced upregulation of miR397 is conserved in rice and other cereal crops. If so, the cold stress–induced upregulation of miR397 expression and miR397-mediated increase in BR sensitivity and grain yield could become another target of genetic manipulation aimed at improving stress tolerance and yield in cereals, particularly in winter wheat and barley.

Another example of the interplay between miRNAs and BR metabolism is illustrated by the miR156-mediated downregulation of the target gene *liguleless1* (*LG1*), which encodes a plant-specific transcription factor involved in leaf development in maize and rice (Aung et al. 2015). LG1 activates the expression of *related to ABI3/VP1 RAV-like1* (*RAVL1*), encoding a transcription factor that positively regulates the expression of genes involved in BR biosynthesis and perception in rice and maize (Je et al. 2010; Tian et al. 2019; Gruszka 2020; Kong et al. 2020). Interestingly, miR156 is highly expressed in rice under stress conditions (salinity and drought) but suppressed under optimal conditions. Stress-induced miR156 expression enhances anthocyanin accumulation and stress tolerance in rice and maize (Cui et al. 2014; Aung et al. 2015; Raza et al. 2023) (Fig. 4). miR156 may take part in both ABA-dependent and ABA-independent drought stress responses (Zhang et al. 2022). Therefore, miR156 could be considered as another target of genetic manipulation aimed at improving the tolerance of cereal crops to environmental stress, particularly salinity and drought stress (Aung et al. 2015).

In maize plants exposed to elevated temperature, the miR156 family showed the highest number of differentially expressed (mostly upregulated) representatives among miRNA families. Moreover, several important regulatory miRNAs involved in the heat stress response have been identified, including miR156 and the above-mentioned miR396 (Zhang et al. 2019a; Zhang et al. 2022). Interestingly, overexpressing miR397, which is involved in the BR signaling-mediated regulation of leaf inclination, resulted in a substantially increased BR sensitivity in maize (Zhang et al. 2013; Peng et al. 2019); this miRNA might also regulate target gene expression to improve tolerance against drought stress (Das and Mondal 2021) (Fig. 4).

In conclusion, further research is needed to fully clarify the interplay between miRNAs and BRs that regulates stress responses in cereals. The artificial miRNA (amiRNA) technique, which is an effective strategy for gene silencing, could be used in future experiments to specifically regulate the expression of target genes in order to determine the functions of miRNAs in BR-dependent responses to abiotic stress (Singroha et al. 2021, 2022). Additionally, some biotechnological tools aimed at modulating miRNA expression, such as regulating amiRNAs and endogenous miRNAs, as well as CRISPR/Cas-mediated miRNA gene editing, could be used to improve BR-dependent stress tolerance in cereals (Shriram et al. 2016; Zhu 2016). Finally, although some initial insight has been gained into the role of the interplay between miRNAs and BRs in regulating stress responses in rice, our understanding of this issue in other cereals is far more limited and requires further research (Singroha et al. 2021).

## Concluding remarks and perspectives

BRs regulate a wide range of developmental and physiological processes in plants. Due to the stimulatory effect of BRs on cell division and elongation, cereal mutants defective in BR biosynthesis or signaling show a dwarf/semi-dwarf, erect stature. For the last several decades, semi-dwarfism and erect plant architecture have been recognized as very important agronomic traits in cereal breeding. More recent reports indicate that the genetic modification (including CRISPR/Cas-mediated gene editing) or biotechnological manipulation (such as overexpression or RNAi-mediated silencing) of BR biosynthesis or signaling genes may lead to the generation of cereal germplasm with improved tolerance to various environmental stresses.

Genes involved in various steps of BR biosynthesis or signaling are currently recognized as potential targets of genetic and biotechnological manipulation to produce cereal germplasm with enhanced adaptability to stress conditions. Nevertheless, further research is needed to expand the list of BR-related genes that could become targets of genetic manipulation in rice and other cereal species. It should also be kept in mind that some members of gene families (particularly involved in BR signaling) may show different (or even opposite) expression patterns during plant responses to stress conditions. Further studies, particularly at the epigenetic, transcriptomic, and metabolic levels, are needed to fully clarify the molecular mechanisms underlying stress responses in cereals. Whether these mechanisms are regulated via interhormonal crosstalk also needs to be verified.

However, during the development of new stress-tolerant cereal cultivars, a balance between improved stress responses and plant yield must be maintained. Thus, further research is needed in order to fine-tune this balance. Importantly, successful approaches (e.g. the overexpression of *OsDGS1* and *OsWRKY53*, the upregulation of miR397) aimed to achieve the balance between improved stress adaptability and the maintenance of plant yield have been applied in rice (Chujo et al. 2007; Zhang et al. 2013; Tian et al. 2017; Wang et al. 2024). However, such approaches should also be applied in other cereals. Another potential option in cereals to achieve the balance between improved stress adaptability and the maintenance of plant development was described in Arabidopsis. In this species, overexpressing the gene encoding the BRI1-LIKE3 (BRL3) receptor homolog, a vascular-enriched member of the BR receptor family including the major BR receptor kinase BRI1, was shown to improve drought resistance through increased accumulation of sugar and osmoprotectant metabolites in roots. The effect overcomes drought-associated growth arrest and is achieved without penalizing overall plant growth (Fabregas et al. 2018). The E286K substitution was identified in the GSK3 kinase in Indian dwarf wheat. This amino acid substitution enhances heat tolerance at the seedling and mature stages of plant development. The improved heat tolerance was achieved via the increased stability of GSK3 and its target protein TaPIF4. Interestingly, the distribution of the E286K variation is currently limited to India and Pakistan (Cao et al. 2024). Therefore, further research should focus on the use of CRISPR/Cas-mediated gene editing to introduce the E286K variation into other wheat cultivars to expand its distribution, as well as in homologous proteins in other cereal species to improve their heat tolerance.

Deeper insight is also needed into miRNA-BR dependencies in cereals. This would allow the interplay between miR396 and BR signaling to be exploited to improve drought tolerance. Similarly, the cold stress–induced upregulation of miR397 expression and the miR397-mediated increase in BR sensitivity and yield may become another target of genetic manipulation to improve stress tolerance and yield, particularly in winter wheat and barley cultivars. This may be achieved through the genetic manipulation of miRNA expression to regulate the expression of target genes in a tissue- or organ-specific manner. Alternatively, new strategies for precise gene editing may allow for CRISPR-mediated miRNA editing to impair miRNA targeting and to fine-tune gene expression (Ahmar et al. 2024).

Functional redundancy (within gene families) and the limitations of genomic redundancy might be overcome using CRISPR-mediated multiknockout of BR-related genes. The CRISPR-mediated editing of promoters could also be used to modulate BR-related gene expression and prevent the binding of negative regulators (Ahmar et al. 2024). Perhaps, the introduction of various genetic modifications and biotechnological manipulations in cereals could be successfully combined with new breeding strategies, such as speed breeding and speed vernalization (Ahmar et al. 2023a, 2024). However, further studies are needed to fully elucidate various aspects of the BR-dependent regulation of the responses of cereals to environmental stress to achieve the above-mentioned objectives, which are of particular importance in the face of ongoing climate change (see “Outstanding questions” section).

## Advances

- Urgent need for efficient development of new stress-tolerant cereal cultivars is fulfilled by semi-dwarf, BR-deficient, or BR-insensitive cereal mutants.
- Genetic manipulations aimed at the development and utilization of new BR-related mutants for the improvement of cereal yield and environmental adaptability are considered as a target for the next biotechnological revolution in agriculture.
- Cereal mutants defective in the BR biosynthesis or response, developed by various mutagenesis and state-of-the-art genome editing methods, show improved tolerance to drought, salt stress, high temperatures, and biotic stresses.
- The newly identified, BR-related cereal mutants may constitute alternatives in the future breeding programs, especially in the face of ongoing climate changes.

## Outstanding questions

- Which mechanisms regulate adaptation of cereals to drought and/or heat stresses exerted during reproductive development?
- To what extent are these mechanisms dependent on the BR homeostasis?
- Is cereal adaptation to environmental stresses regulated on the basis of BR-dependent epigenetic mechanisms of DNA methylation and/or histone modifications?
- Which genes participating in the regulation of BR homeostasis (particularly the BR signaling) should be selected as optimal targets of the genetic manipulations in order to achieve the broadest range of tolerance against the environmental stresses with the lowest potential risk of side effect on plant yield?
- What is the molecular mechanism of the increased thermotolerance observed in the wheat mutant with the gain-of-function mutation of the *TaGSK3* gene?

## Data Availability

No new data were generated or analysed in support of this research.

## Dive Curated Terms

The following phenotypic, genotypic, and functional terms are of significance to the work described in this paper:

AGO1 Gramene: AT1G48410
AGO1 Araport: AT1G48410

BRL3 Gramene: AT3G13380
BRL3 Araport: AT3G13380

PIF4 Gramene: AT2G43010
PIF4 Araport: AT2G43010

ABA Gramene: Abscisic acid
ABA Araport: Abscisic acid

CPD Gramene: AT5G05690
CPD Araport: AT5G05690

BRI1 Gramene: AT4G39400
BRI1 Araport: AT4G39400

BAK1 Gramene: AT4G33430
BAK1 Araport: AT4G33430

DGS1 Gramene: Os03g0706900
DGS1 Araport: Os03g0706900

WRKY53 Gramene: AT4G23810
WRKY53 Araport: AT4G23810

OsBRI1 Gramene: Os01g0718300
OsBRI1 Araport: Os01g0718300
